# Early Response of Antimicrobial Resistance and Virulence Genes Expression in Classical, Hypervirulent, and Hybrid hvKp-MDR *Klebsiella pneumoniae* on Antimicrobial Stress

**DOI:** 10.3390/antibiotics11010007

**Published:** 2021-12-22

**Authors:** Anastasiia D. Fursova, Mikhail V. Fursov, Evgenii I. Astashkin, Tatiana S. Novikova, Galina N. Fedyukina, Angelina A. Kislichkina, Irina A. Alexandrova, Olga N. Ershova, Ivan A. Dyatlov, Nadezhda K. Fursova

**Affiliations:** 1 State Research Center for Applied Microbiology and Biotechnology, Territory “Kvartal A”, 142279 Obolensk, Moscow Region, Russia; anfursova06@gmail.com (A.D.F.); mikhail.fursov88@gmail.com (M.V.F.); ast.ev@mail.ru (E.I.A.); positifka.15@yandex.ru (T.S.N.); galafed@mail.ru (G.N.F.); angelinakislichkina@yandex.ru (A.A.K.); dyatlov@obolensk.org (I.A.D.); 2 National Medical Research Center of Neurosurgery Named after Academician N.N. Burdenko, 125047 Moscow, Russia; ialexandrova@nsi.ru (I.A.A.); oershova@nsi.ru (O.N.E.)

**Keywords:** Klebsiella pneumoniae, sequence type, capsular type, MDR, hvKp, hybrid hvKp-MDR, resistance genes, virulence genes, gene expression, qPCR

## Abstract

*Klebsiella pneumoniae* is an increasingly important hospital pathogen. Classical *K. pneumoniae* (cKp) and hypervirulent *K. pneumoniae* (hvKp) are two distinct evolutionary genetic lines. The recently ongoing evolution of *K. pneumoniae* resulted in the generation of hybrid hvKP-MDR strains. *K. pneumoniae* distinct isolates (*n* = 70) belonged to 20 sequence types with the prevalence of ST395 (27.1%), ST23 (18.6%), ST147 (15.7%), and ST86 (7.1%), and 17 capsular types with the predominance of K2 (31.4%), K57 (18.6%), K64 (10.0%), K1 (5.7%) were isolated from patients of the Moscow neurosurgery ICU in 2014–2019. The rate of multi-drug resistant (MDR) and carbapenem-resistant phenotypes were 84.3% and 45.7%, respectively. Whole-genome sequencing of five selected strains belonging to cKp (ST395^K47^ and ST147^K64^), hvKp (ST86^K2^), and hvKp-MDR (ST23^K1^ and ST23^K57^) revealed *bla*_SHV_, *bla*_TEM_, *bla*_CTX_, *bla*_OXA-48_, and *bla*_NDM_ beta-lactamase genes; *acr*, *oqx*, *kpn*, *kde*, and *kex* efflux genes; and *K. pneumoniae* virulence genes. Selective pressure of 100 mg/L ampicillin or 10 mg/L ceftriaxone induced changes of expression levels for named genes in the strains belonging to cKp, hvKp, and hybrid hvKp-MDR. Obtained results seem to be important for epidemiologists and clinicians for enhancing knowledge about hospital pathogens.

## 1. Introduction

*Klebsiella pneumoniae* is an increasingly important hospital pathogen causing a wide range of infections including urinary tract infections, pneumonia, bacteremia, and liver abscesses. In severe clinical cases, it can also lead to multiple organ failure, or even death [1]. Two different evolutionary genetic lines, classical *K. pneumoniae* (cKp) and hypervirulent *K. pneumoniae* (hvKp), were described and are both global pathogens [2]. Most multidrug-resistant (MDR) *K. pneumoniae* strains belong to particular clones (ST11, ST395, ST147, etc.) producing beta-lactamases in combination with other functional classes of resistance determinants [3]. Hypervirulent *K. pneumoniae* were attributed to sequence types ST23, ST86, ST65, etc., and capsular types K1, K2, K57, K20, etc. [4]. Virulence determinants of hvKp include siderophore systems for iron acquisition, increased capsule production, and the colibactin toxin commonly located on virulence plasmids [5].

Recent studies have shown the ongoing evolution of *K. pneumoniae* resulting in the generation of hybrid hvKp-MDR strains. Mechanisms for the emergence of such strains can be a result of acquiring hypervirulent plasmids by cKp [6], acquiring MDR plasmids by hvKp [7], and acquiring hybrid virulence-MDR plasmids [8]. However, the relative expression of resistance and virulence genes in bacteria of cKp, hvKp, and hybrid hvKp-MDR evolutionary branches is poorly studied.

This study aimed to determine the genetic lines of *K. pneumoniae* strains collected in Moscow neurosurgery ICU in 2014–2019, to identify resistance and virulence genes in their cells, and to estimate relative expression levels of such genes in selected strains belonging to epidemiology significant genetic lines cKp (ST395^K47^ and ST147^K64^), hvKp (ST86^K2^), and hybrid hvKp-MDR (ST23^K1^ and ST23^K57^).

## 2. Results

### 2.1. Bacterial Isolates and Clinical Data

*K. pneumoniae* caused about 28% among the agents of nosocomial infections in neurosurgery ICU during the period from January 2014 to May 2019. The incidence rates of *K. pneumoniae* infections were 8.0 per 100 patient infections of the central nervous system, 4.3/100 of bloodstream infections, 26.3/100 of respiratory infections, and 25.3/100 of urinary tract infections [9,10]. A total of 545 *K. pneumoniae* clinical isolates were collected from 283 patients in this period, including those isolated from the respiratory system (*n* = 271), urine (*n* = 166), the nervous system (*n* = 41), blood (*n* = 36), surgical wounds (*n* = 27), and other (*n* = 4).

### 2.2. K. pneumoniae Sequence Types and Capsular Types

**Figure 1:**
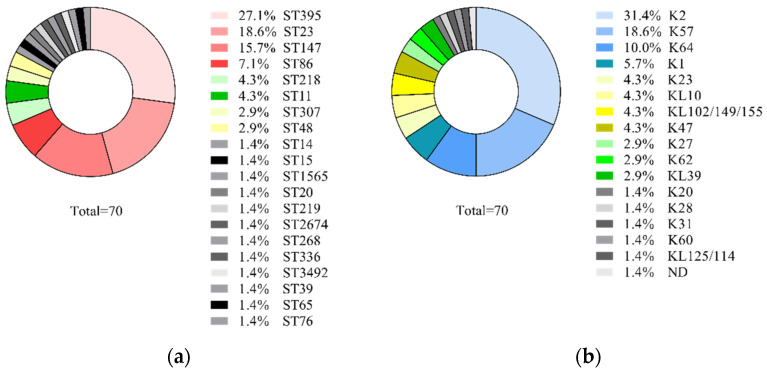
Prevalence of *K. pneumoniae* genetic lines among 70 isolates collected in Neuro-ICU in 2014–2019: (**a**) sequence types; (**b**) capsular types.

Seventy non-duplicate isolates selected from the collection of 545 isolates were characterized by sequence types and capsular types. These isolates were collected from the respiratory system (*n* = 34), urine (*n* = 19), the nervous system (*n* = 8), blood (*n* = 6), and surgical wounds (*n* = 3). As a result, 20 sequence types were identified, the majority of them were ST395, ST23, ST147, and ST86, and a total of 17 capsular types were identified. Predominant K-types were K2, K57, K64, and K1 (Figure 1).

### 2.3. Susceptibility to Antimicrobials and Resistance Genes

**Figure 2:**
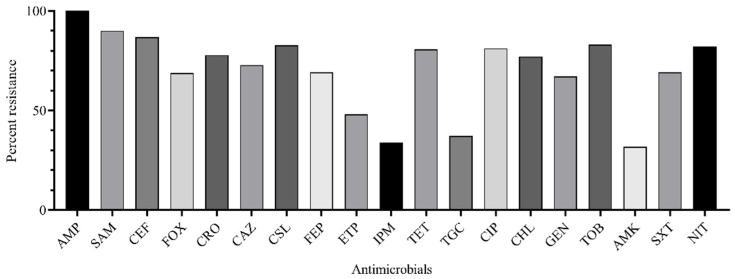
Rate of *K. pneumoniae* isolates (*n* = 70) resistant to antimicrobials: AMP, ampicillin; SAM, ampicillin-sulbactam; CXM, cefuroxime; FOX, cefoxitin; CRO, ceftriaxone; CAZ, ceftazidime; CSL, cefepime; FEP, cefepime; ETP, ertapenem; IPM, imipenem; TET, tetracycline; TGC, tigecycline; CIP, ciprofloxacin; CHL, chloramphenicol; GEN, gentamicin; TOB, tobramycin; AMK, amikacin; SXT, trimethoprim-sulfamethoxazole; NIT, nitrofurantoin.

It was shown that major *K. pneumoniae* isolates were resistant to ampicillin (100.0%), ampicillin-sulbactam (90.0%), cefuroxime (86.8%), cefoxitin (68.8%), ceftriaxone (77.8%), ceftazidime (72.8%), cefoperazone-sulbactam (82.7%), cefepime (69.2%), ertapenem (48.1%), tetracycline (80.7%), ciprofloxacin (81.2%), chloramphenicol (77.0%), gentamicin (67.1%), tobramycin (83.0%), trimethoprim-sulfamethoxazole (69.2%), and nitrofurantoin (82.2%). Fewer resistant isolates were found for imipenem (33.8%), tigecycline (37.2%), and amikacin (31.8%) (Figure 2).

The frequency of the multi-drug resistant (MDR) phenotype was 84.3%, with the carbapenem-resistant phenotype consitituting 45.7%. *K. pneumoniae* isolates carried beta-lactamase genes *bla*_SHV_ (100.0%), *bla*_CTX-M_ (74.2%), *bla*_TEM_ (51.4%), *bla*_OXA-48_ (40.0%), *bla*_NDM_ (11.4%), class 1 (37.6%) and 2 (2.1%) integrons, and porin protein gene *ompK36* (91.5%). Both *bla*_OXA-48_ and *bla*_NDM_ carbapenemase genes were detected in four (5.7%) isolates. Beta-lactamase genes *bla*_KPC_, *bla*_VIM_, and *bla*_IMP_ were not detected (Table S1).

### 2.4. K. pneumoniae Virulence Genes

Eight virulence genes were detected in 70 *K. pneumoniae* isolates: *rmpA* (34.2%), *aer* (21.4%), *kfu* (17.1%), *uge* (85.7%), *wabG* (100.0%), *fimH* (97.1%), *allS* (94.3%), and *allR* (5.7%). Twelve virulence gene combinations were identified. The most prevalent combination was *uge*+*wabG*+*fimH*+*allS* (51.4% isolates), followed by *rmpA*+*aer*+*uge*+*wabG*+*fimH*+*allS* (11.4%), *uge*+*wabG+kfu*+*fimH*+*allS* (7.1%), *rmpA*+*wabG*+*fimH*+*allS* (5.7%), and *rmpA*+*aer*+ +*uge*+*wabG+kfu*+*fimH*+*allR* (5.7%). The rarest combinations were *wabG*+*fimH*+*allS* (4.3%), *rmpA+uge*+*wabG*+*fimH*+*allS* (4.3%), *uge*+*wabG*+*allS* (2.8%), *rmpA+aer*+*wabG*+*fimH+allS* (2.8%), *rmpA+wabG+kfu*+*fimH+allS* (1.4%), *rmpA*+*aer*+*uge*+*wabG+kfu*+*fimH*+*allS* (1.4%), and *rmpA*+*wabG+kfu*+*fimH*+*allS* (1.4%) (Table S2).

### 2.5. Hypermucoviscousity Phenotype 

Phenotype of hypermucoviscousity was detected for 11 of 70 isolates (15.7%) collected from the respiratory system (*n* = 9) and urine (*n* = 2), belonging to genetic lines ST23^K1^ (*n* = 4), ST86^K2^ (*n* = 3), ST23^K57^ (*n* = 2), ST65^K2^ (*n* = 1), and ST218^K57^ (*n* = 1). Isolates of ST23^K1^ carried *rmpA*+*aer*+*uge*+*wabG+kfu*+*fimH*+*allR*; isolates of ST86^K2^ and ST218^K57^ harbored *rmpA*+*aer*+*uge*+*wabG*+*fimH*+*allS*; isolates of ST23^K57^ carried two combinations, *rmpA+uge*+*wabG*+*fimH*+*allS* and *rmpA+wabG+kfu*+*fimH+allS*; and isolates of ST65^K2^ carried *rmpA*+*aer*+*uge*+*wabG+kfu*+*fimH*+*allS* virulence gene combinations. All 11 hypermucoviscous isolates carried *rmpA*, *wabG*, and *fimH* genes. Major isolates of hypermucoviscousity-positive isolates were MDR (*n* = 6), resistant to 3–7 antimicrobial functional groups (beta-lactams, aminoglycosides, tetracyclines, phenicols, fluoroquinolones, sulfonamides, nitrofurans). Among them, two isolates were CR. The remaining isolates were resistant to ampicillin only. As a result, there was no revealed correlation between the hypermucoviscousity phenotype and genetic line or the antimicrobial phenotype or virulence gene profiles (Tables S1 and S2).

### 2.6. Whole-Genome Sequencing

Five clinical isolates belonging to epidemically significant *K. pneumoniae* genetic lines, cKp (ST395^K47^ and ST147^K64^), hvKp (ST86^K2^), and hvKp-MDR (ST23^K1^ and ST23^K57^), were analyzed by whole-genome sequence and quantitative analyses of antimicrobial resistance and virulence gene expression. One of these strains (B2523/18) was resistant to AMP and carried only one beta-lactamase gene of the *bla*_SHV_ type. In contrast, the other four strains that belonged to the MDR category were resistant to 10–18 antimicrobials. The latter strains carried beta-lactamase genes of *bla*_SHV_, *bla*_TEM_, *bla*_CTX-M_, *bla*_OXA-48_, and *bla*_NDM_ types and the resistance determinants for other antimicrobial groups in the genomes: aminoglycosides, fosfomycin, streptogramins, phenicols, quinolones, sulfonamide, tetracyclines, sulfonamides, macrolides, and rifamycins, as well as genes coding 4–5 efflux pumps and 1–4 genes of heavy metal resistance. The following genetic determinants of *K. pneumoniae* virulence were identified in the genomes: *peg-344* (*n* = 3), *rmpA* (*n* = 3), *rmpA2* (*n* = 1), *iroB* (*n* = 2), *iroN* (*n* = 2), *iroD* (*n* = 2), *uge* (*n* = 5), *wabG* (*n* = 5), *kfu* (*n* =1 ), *fimH* (*n* = 5), *allR* (*n* = 1), *irp* (*n* = 4), *iuc* (*n* = 3), *entB* (*n* = 3), *iut* (*n* = 4), *mrk* (*n* = 5), *ybt* (*n* = 4), *fyu* (*n* =4 ), *treC* (*n* = 5), *celB* (*n* = 5), and *ureA* (*n* = 5) (Table 1).

### 2.7. Relative mRNA Levels of Antimicrobial Resistance and Virulence Genes in K. pneumoniae Cells

**Figure 3:**
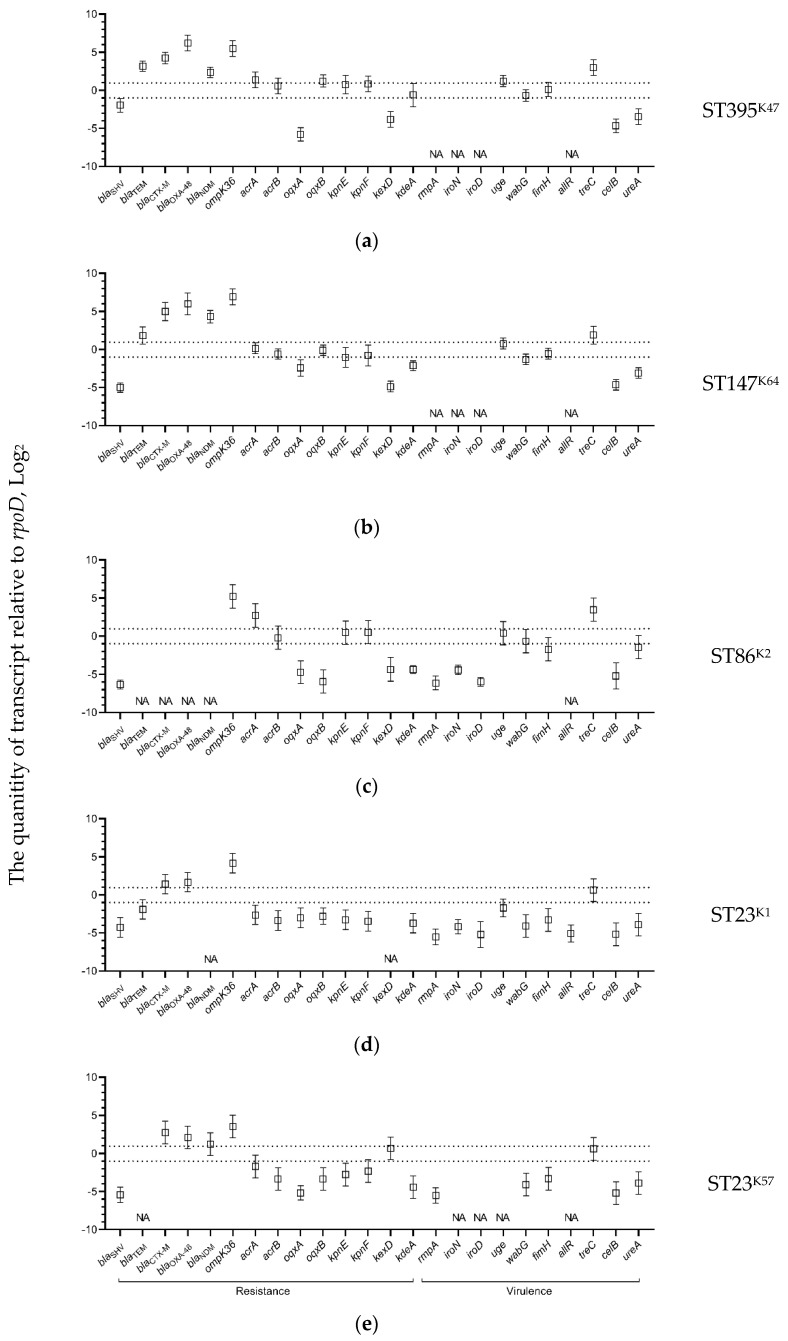
Expression levels of K. pneumoniae antimicrobial resistance and virulence genes detected in the strains: (**a**) B-185/19; (**b**) B-16K/19; (**c**) B-2523/18; (**d**) B-2580/14; and (**e**) B-1261/19 detected by qPCR compared with rpoD gene as a reference. Expression levels for three replicates along with the standard error of the mean are shown on the plots; the area of no significant differences with the expression of the reference gene (from minus 1 to 1) are circumscribed by dotted lines; NA, not acceptable.

The expression levels of the resistance genes in all *K. pneumoniae* strains during growth without selective pressure of antimicrobials were different: the chromosomal beta-lactamase genes *bla*_SHV_ were transcribed significantly lower compared with those of the reference gene *rpoD*. In contrast, other beta-lactamase genes (*bla*_TEM,_ *bla*_CTX-M_, *bla*_OXA-48_, and *bla*_NDM_), as well as porin gene *ompK*36 were transcribed at higher levels compared with the reference gene, with the exception of *bla*_TEM_ in the hvKp-MDR strain of ST23^K1^ which exhibited lower expression. The efflux pump genes and the virulence genes were expressed mostly at the same or lower levels compared with the *rpoD* gene, with the exception of two virulence genes: *treC* in the strains of ST395^K47^, ST147^K64^, ST23^K1^, and ST23^K57^; and *celB* in the strain of ST86^K2^ (Figure 3).

### 2.8. Fold Change of Expression Levels of K. pneumoniae Antimicrobial Resistance and Virulence Genes under AMP and CRO Selective Pressure

**Figure 4:**
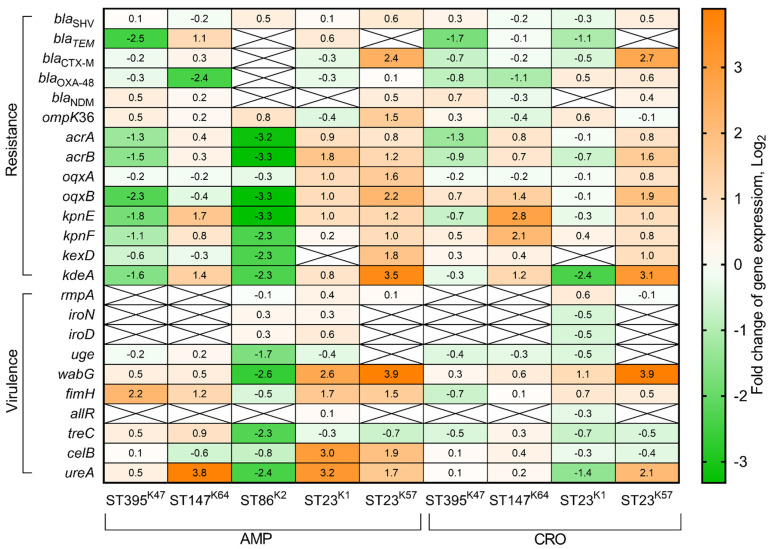
Heatmap of changes in gene expression levels of *K. pneumoniae* resistance and virulence genes under ampicillin (100 mg/L) and ceftriaxone (10 mg/L) selective pressure during 90 min in LB medium in comparison with expression levels of these genes in LB medium without antibiotics. Values of each cell represent log_2_ of gene expression fold changes (fold changes ≥1 or ≤−1 are significant).

We analyzed gene expression patterns of antimicrobial resistance and virulence genes of *K. pneumoniae* strains belonging to different genetic lines in the presence of 100 mg/L AMP. The MDR strain of ST395^K47^ demonstrated upregulation of the *fimH* (4.6-fold) gene, and downregulation of the *bla*_TEM_ (5.5-fold), *acrA* (2.4-fold), *acrB* (2.8-fold), *oqxB* (4.8-fold), *kpnE* (3.5-fold), *kpnF* (2.0-fold), and *kdeA* (3.0-fold) genes. Another MDR strain of ST147^K64^ was characterized by the upregulation of the *bla*_TEM_ (2.2-fold), *kpnE* (3.2-fold), *kdeA* (2.7-fold), *fimH* (2.3-fold), and *ureA* (14.0-fold) genes, and downregulation of the *bla*_OXA-48_ (5.3-fold) gene. The hvKp strain of ST86^K2^ demonstrated upregulation of only one gene, *iroN* (4.1-fold), and downregulation of 13 genes: *bla*_SHV_ (2.6-fold), *acrA* (9.0-fold), *acrB* (10.0-fold), *oqxB* (10.1-fold), *kpnE* (9.9-fold), *kpnF* (5.0-fold), *kexD* (4.9-fold), *kdeA* (4.8-fold), *iroD* (4.0-fold), *uge* (3.2-fold), *wabG* (6.2-fold), *treC* (4.8-fold), and *ureA* (5.3-fold). The hybrid hvKp-MDR strain of ST23^K1^ showed upregulation of the *acrB* (3.4-fold), *oqxA* (2.1-fold), *oqxB* (2.1-fold), *wabG* (6.2-fold), *fimH* (3.2-fold), *celB* (8.0-fold), and *ureA* (8.9-fold) genes. The second hvKp-MDR strain of ST23^K57^ expressed upregulation of the *bla*_TEM_ (4.1-fold), *bla*_CTX-M_ (5.3-fold), *ompK36* (2.9-fold), *acrB* (2.3-fold), *oqxA* (2.9-fold), *oqxB* (4.6-fold), *kpnE* (2.3-fold), *kpnF* (2.0-fold), *kexD* (3.2-fold), *kdeA* (11.7-fold), *uge* (3.4-fold), *wabG* (14.5-fold), *fimH* (2.9-fold), *celB* (3.7-fold), and *ureA* (3.1-fold) genes (Figure 4).

Additionally, we estimated fold changes of expression levels for the above listed genes in response to 10 mg/L CRO conditions. The MDR strain of ST395^K47^ demonstrated downregulation of the *bla*_TEM_ (3.3-fold) and *acrA* (2.4-fold) genes. The MDR strain of ST147^K64^ was characterized by upregulation of the *oqxB* (2.7-fold), *kpnE* (7.0-fold), *kpnF* (4.4-fold), *kdeA* (2.3-fold) genes, and downregulation of the *bla*_OXA-48_ (2.1-fold) gene. The hybrid hvKp-MDR strain of ST23^K1^ showed upregulation of the *fimH* (2.1-fold) gene, and downregulation of the *bla*_TEM_ (2.1-fold), *kdeA* (5.1-fold), and *ureA* (2.7-fold) genes. The second hybrid hvKp-MDR strain of ST23^K57^ expressed upregulation of the *bla*_SHV_ (2.3-fold), *bla*_TEM_ (2.4-fold), *bla*_CTX-M_ (6.3-fold), *acrB* (3.0-fold), *oqxB* (3.8-fold), *kpnE* (2.1-fold), *kdeA* (8.6-fold), *wabG* (14.8-fold), and *ureA* (4.2-fold) genes, and no genes were downregulated (Figure 4).

## 3. Discussion

*K. pneumoniae* was one of the major nosocomial pathogens in a Moscow neurosurgery ICU during the period from January 2014 to May 2019, causing about 28% infections including those of the central nervous system, bloodstream, respiratory tract, and urinary tract. This rate was similar to those in the Multispecialty Hospital, Riga, Latvia in 2017–2020 (16–20%) [11], and significantly lower than those reported from 15 China centers in 2012–2016 (52.4%) [12].

Non-duplicate 70 *K. pneumoniae* isolates collected from 62 patients were attributed to specific genetic lines, virulence, and antimicrobial resistance genotypes. Single isolates were collected from 54 patients and two isolates from eight patients. Double isolates were studied from one patient in a case of their differences in ST (Patients 19, 32, 36, 46, and 48), K-type (Patient 35), and antimicrobial resistance genes profiles (Patients 4 and 39) (Table S1). Two isolates collected from the trachea and urine of the Patient 4 attributed to ST147^K64^ carried (*bla*_TEM_+*bla*_SHV_+*bla*_CTX-M_+*bla*_OXA-48_+*bla*_NDM_) and (*bla*_SHV_+*bla*_OXA-48_) beta-lactamase genes, respectively. Two isolates obtained from the trachea of Patient 39 belonged to ST23^K57^ (*bla*_TEM_+*bla*_SHV_+*bla*_CTX-M_) and (*bla*_SHV_+*bla*_CTX-M_+*bla*_OXA-48_) beta-lactamase genes, respectively. Different antimicrobial resistance gene profiles of *K. pneumoniae* named isolates possibly indicate the evolution events in the patient’s body as described previously [13].

Twenty STs and 17 K-types were identified. The most prevalent sequence types were ST395 (27.1%), ST23 (18.6%), ST147 (15.7%), and ST86 (7.1%). The remaining 16 STs were each represented at ≤ 4.3%. The dominant STs were described previously as *K. pneumoniae* genetic lines common for classical *K. pneumoniae* (cKp) (ST395 and ST147) and hypervirulent *K. pneumoniae* (hvKp) (ST23 and ST86). ST395 was reported as prevalent for cKp-MDR strains in studies from Poland, France, Italy, and Russia [14,15,16,17]. ST147 has been estimated as Hight-Risk-Clone of cKp distributed worldwide, causing serious infections and associated with polyresistance [18]. *K. pneumoniae* of ST23 and ST86 were described previously as hvKp causing bacteremia, sepsis, and liver abscess in India, France, Taiwan, and Russia [19,20,21,22]. The prevalent K-types in our study were K2 (31.4%), K57 (18.6%), K64 (10.0%), and K1 (5.7%); the remaining of the 12 K-types were each present at a frequency of ≤ 4.3%. Among them, K1, K2, and K57 were identified in many studies as specific capsular types for hvKp [23]. *K. pneumoniae* isolates of K64 commonly were recognized as cKp, but recently some isolates of K64 were described as hybrid hvKp-MDR due to acquiring the hvKp virulence plasmids [24]. Moreover, many reports have been published in the last decade concerning hvKp-MDR hybrids generated on the base of cKp ST11^K20/K64^, ST395^K2^, as well as hvKp ST23^K1^ and ST86^K2^ [25,26,27,28,29]. Major (84.3%) *K. pneumoniae* isolates in our study were MDR with resistance to carbapenems for 45.7% isolates. Such high MDR rates were reported from China and Iran [30,31]. The prevalence of beta-lactamase genes *bla*_CTX-M_ in our study was 74.2%, carbapenemase genes *bla*_OXA-48_ and *bla*_NDM_ at 40.0% and 11.4%, respectively, similar to the prevalence of these genes published previously [31]. Hypermucoviscous isolates identified in this study (*n* = 11) were attributed to ST23^K1^, ST86^K2^, ST23^K57^, ST65^K2^, and ST218^K57^ genetic lines. All of them carried the *rmpA* gene coding the major virulence-associated factor in hvKP isolates [32], although not all *rmpA*-positive isolates in this study were hypermucoviscousity-positive, which is in agreement with a previously published report [22]. Hypermucoviscous isolates were characterized in our previous study as hypervirulent in the mouse model [22]. The rate of MDR *K. pneumoniae* among hypermucoviscousity-positive isolates in this study was 6/11, which is consistent with the modern trend for the appearance of hybrid hvKp-MDR genetic lines [8,33,34].

Five *K. pneumoniae* strains belonging to prevalent STs and K-types (cKp, ST395^K47^, and ST147^K64^; hvKp, ST86^K2^; and hybrid hvKp-MDR, ST23^K1^, and ST23^K57^) were selected for further comparative study of whole-genome sequences, antimicrobial resistance phenotypes, hypermucoviscosity, and altered expression levels of virulence and resistance genes in response to beta-lactams effect (AMP and CRO). It was shown that *K. pneumoniae* isolates belonging to ST86^K2^, ST23^K1^, and ST23^K57^ demonstrated hypermucoviscosity phenotype in contrast with isolates of ST395^K47^ and ST147^K64^, which confirmed the virulent phenotype of three isolates. All *K. pneumoniae* isolates carried *bla*_SHV_ genes, including extended-spectrum beta-lactamase (ESBL) variant *bla*_SHV-12_, broad-spectrum variants *bla*_SHV-28_ and *bla*_SHV-33_, and narrow-spectrum variants *bla*_SHV-67_ and *bla*_SHV-190_. These alleles of *bla*_SHV_ genes were previously described in Portugal, Turkey, China, Russia, and Spain [22,35,36]. Three *K. pneumoniae* isolates (ST395^K47^, ST147^K64^, and ST23^K1^) carried the *bla*_TEM-1B_, *bla*_CTX-M-15_, and *bla*_OXA-1_ genes. It was reported that these genes were horizontally transferred by the IncFIA-FIB-FII and IncHI2 plasmids [37,38]. One isolate (ST23^K57^) carried the *bla*_CTX-M-55_ and *bla*_OXA-1_ genes; such gene combination was reported previously from China [39]. It should be noted that MDR isolates of ST395^K47^ and ST147^K64^ additionally carried two carbapenemase genes, i.e., bla_OXA-48_ and bla_NDM-1_. Previously, it was reported that *K. pneumoniae* clinical isolates of ST395 and ST147 harbored *bla*_NDM-5_ and *bla*_OXA-181/232_ in Nepal and that ST11 harbored *bla*_NDM-1_ and *bla*_OXA-48_ in Greece [33,40]. Of greatest interest are *K. pneumoniae* isolates belonging to the hvKp evolutionary branch, which acquired the resistance genes and became a hybrid hvKp-MDR. In our study, a hvKp-MDR isolate of ST23^K1^ carried simultaneously *bla*_CTX-M-15_ and *bla*_OXA-48_ genes, similar to a recently published study [34]. Moreover, another hvKp-MDR isolate of ST23^K57^ carried not only the *bla*_CTX-M-55_ and *bla*_OXA-48_ genes but additionally the *bla*_NDM-1_ gene. This is the first report describing *K. pneumoniae* of ST23^K57^ genetic-line-acquired cefalosporinase gene *bla*_CTX-M-55_, and two carbapenemase genes *bla*_OXA-48_ and *bla*_NDM-1_, which is particularly alarming. The incidence of high-risk clone ST383 carrying *bla*_CTX-M-14b_ gene and two carbapenemase genes *bla*_NDM-1_ and *bla*_OXA-48_ combining both resistance and virulence elements was recently published [8] as well as the incidence of ST147-carrying *bla*_CTX-M-15_, *bla*_NDM_, and *bla*_OXA-181_ genes [41]. Additionally, we detected the efflux pump genes (*acrA, acrB, oqxA, oqxB, kpnE, kpnF, kdeA,* and *kexD*) because of their clinical significance for *K. pneumoniae* beta-lactam resistance presented recently [41]. In our study, one strain of ST23^K1^ carried four efflux pump genes: *acr* and *oqx* of RND-type systems, *kde* of MATE-type, and *kpn* of SMF-type. The rest of the four strains carried five efflux pumps: *acr*, *oqx*, *kde*, *kpn*, and additionally the *kex* gene of the RND-type efflux system. The same efflux pump genes were detected recently in *K. pneumoniae* clinical isolates, which exhibited co-resistance to beta-lactams and aminoglycosides, glycopeptides, fluoroquinolones, and tetracyclines in India [41]. Interestingly, the efflux pumps were associated with bacterial virulence, namely biofilm formation [42].

It is known that multiple biomarkers have been shown to predict hvKp isolates: *peg-344*, *iroB*, *iucA*, plasmid-encoded *rmpA,* and *rmpA2* and quantitative siderophore production (*entB* and *ybtS*) [43]. In this study, these genes were detected only in the genomes of hvKp isolates of ST86^K2^, ST23^K1^, and ST23^K57^. In contrast, *K. pneumoniae* virulence genes (*uge, wabG, fimH, mrk,* *treC, cellB,* and *ureA*), common for both hvKp and cKp, were detected in all five isolates. This is in agreement with recently published data [44]. Two strains of ST23^K1^ and ST23^K57^ are characterized as hybrid hvKp-MDR. Thus, the data obtained in this study indicate the ongoing formation of hybrid *K. pneumoniae* on the base of the ST23 genetic line, which was already defined in the last decade [7,22,35,45].

We estimated the basal expression levels of *K. pneumoniae* resistance and virulence genes at non-selective conditions *in vitro* and the fold change of expression levels in presence of AMP and CRO. It was shown that ESBL gene *bla*_SHV-12_ expressed ~4-fold higher in the MDR strain of ST395^K47^ than *bla*_SHV_-type genes coding broad-spectrum and narrow-spectrum beta-lactamases in the remaining *K. pneumoniae* strains. This is in agreement with previously reported data that ESBL variants of the *bla*_SHV-12_ gene expressed higher than non-ESBL variants [46]. These genes did not change their expression after 90 min growing at 100 mg/L AMP or 10 mg/L CRO.

Beta-lactamase genes *bla*_TEM,_ *bla*_CTX-M_, *bla*_OXA-48_, and *bla*_NDM_ and porin gene *ompK*36 were expressed at higher levels, with the exception of *bla*_TEM_ in the hvKp-MDR strain of ST23^K1^. Notably, the expression levels of the beta-lactamase (*bla*_TEM_ and *bla*_CTX-M_) and carbapenemase (*bla*_OXA-48_, and *bla*_NDM_) genes were higher in cKp-MDR strains than those in hybrid hvKp-MDR strains. Possibly, the reason for this observation is the higher metabolic load in *Klebsiella* cells producing resistance and virulence factors simultaneously. Growing at AMP conditions for 90 min induced upregulation of *bla*_TEM_ gene expression in cKp-MDR of ST147^K64^ (2.1-fold), as well as the *bla*_CTX-M_ (5.3-fold) and *ompK*36 (2.8-fold) genes in hvKp-MDR of ST23^K57^, but downregulation of *bla*_TEM_ gene expression in cKp-MDR of ST395^K47^ and the *bla*_OXA-48_ gene in cKp-MDR of ST147^K64^. In contrast, growing at CRO conditions induced upregulation of the *bla*_CTX-M_ gene in hvKp-MDR of ST23^K57^ (6.5-fold) and downregulation of the *bla*_TEM_ gene in cKp-MDR of ST395^K47^ (3.3-fold), in hvKp-MDR of ST23^K1^ (2.1-fold), and the *bla*_OXA-48_ gene in cKp-MDR of ST147^K64^ (2.1-fold).

The basal level of efflux pump gene expression was very different for cKp, hvKp, and hybrid hvKp-MDR strains. In cKp-MDR strains, 5–6 efflux genes were expressed on the same level as reference gene *ropD*, and 2–3 genes were lower than the reference. In the hvKp strain, one efflux gene expression was higher, three genes were expressed at the same level, and four genes were lower than the reference gene. In contrast, major efflux genes in hybrid hvKp-MDR strains were expressed lower than the *rpoD* gene, and one gene in the strain of ST23^K57^ was expressed on the same level as the reference. Interestingly, the previously described expression of the *arcB* efflux gene showed upregulation of this gene in carbapenem-resistant *K. pneumoniae* strains compared with non-resistant ones [47]. The relatively high expression level of efflux genes in cKp and hvKp strains indicates the importance of efflux pumps for virulence of both *Klebsiella* evolutionary branches that are consistent with previous reports [48]. It is known that efflux pumps use different antimicrobials as substrates. Our results suggest that the change in efflux gene expression in certain *K. pneumoniae* genetic lines may reflect differences in bacterial surface structures in particular K-types: downregulation in the strains of K47 and K2, and upregulation in the strains of K1 and K57 in response to AMP; and upregulation in the strains of K64 and K57 in response to CRO (Figure 4).

It was shown in our study that transcripts of *K. pneumoniae* virulence genes common for both the cKp and hvKp evolutionary branches (*uge*, *wabG*, *fimH*, and *treC*) were present at higher levels in the cKp strains of ST395^K47^, ST147^K64^, and hvKP ST86^K2^ than in the hybrid hvKp-MDR strains of ST23^K1^ and ST23^K57^. In conditions containing 100 mg/L AMP, these genes were upregulated in cKp and hybrid hvKp-MDR strains (*fimH* 2.3-4.6-fold) and *wabG* in hvKp-MDR strains (6.1–14.9-fold), while downregulated in the hvKp strain (*uge*, *wabG*, and *treC,* 3.2-4.9-fold). In conditions with 10 mg/L CRO, only the *wabG* gene was upregulated in hybrid hvKp-MDR strains (2.3-14.9-fold). The expression levels of the remaining virulence genes common for cKp and hvKp (*celB* and *ureA*), as well as common for only hvKp (*rmpA*, *iroN*, *iroD*, and *allR*), were approximately equal in all studied strains at non-selective conditions. The *celB* gene expression at AMP medium was upregulated in hvKp-MDR strains (3.7–8.0-fold); the *ureA* gene expression was upregulated in the cKp strain of ST147^K64^ (13.9-fold) and hvKp-MDR strains of ST23^K1^ (9.2-fold) and ST23^K57^ (3.2-fold). It was detected that CRO induced upregulation of the *ureA* gene in a hvKp-MDR strain of ST23^K57^ (4.3-fold) and downregulation of this gene in a hvKp-MDR strain of ST23^K1^ (2.6-fold). Expression levels of virulence genes common for hvKp strains were not changed under selective pressure generated by AMP or CRO in the growth media (Figure 4).

In summary, expression levels of antimicrobial resistance and virulence genes were characteristic for *K. pneumoniae* of different certain genetic lines. Selective pressure by sub-inhibitory concentrations of ampicillin or ceftriaxone induced differential upregulation or downregulation of these genes depending on the strain belonging to classical cKp, hypervirulent hvKp, or hybrid hvKp-MDR evolutionary branches. Results obtained in this study may be fruitful for future studies of evolution, the spread of antimicrobial and virulence genetic determinants, and the clinical impact of *K. pneumoniae* genetic lines.

## 4. Materials and Methods

### 4.1. Bioethical Requirements and Patients

*K. pneumoniae* isolates were collected from the patients of the neuro-intensive care unit (Neuro-ICU) in a specialized Neurosurgical Hospital in Moscow, Russia. Following the requirements of the Russian Federation Bioethical Committee, each patient signed informed voluntary consent to treatment and laboratory examination. The materials used in the study did not contain the personal data of patients.

### 4.2. Bacterial Isolates, Identification, and Growth Conditions

Seventy *K. pneumoniae* isolates were collected from the respiratory system, blood, urine, cerebrospinal fluid, and wounds of 62 patients of the neuro-ICU. Bacteria identification was performed using by a Vitek-2 Compact instrument (BioMérieux, Paris, France) and a MALDI-TOF Biotyper (Bruker Daltonics, Bremen, Germany). Bacteria were grown at 37 °C with agitation on Luria-Bertani broth (Difco Laboratories, Detroit, MI, USA) and Muller-Hinton broth (Becton Dickinson, Franklin Lakes, NJ, USA). Bacterial isolates were stored in 20% glycerol at minus 80 °C.

### 4.3. Susceptibility to Antimicrobials

Minimal inhibitory concentrations (MICs) of ampicillin (AMP), ampicillin-sulbactam (SAM), cefuroxime (CXM), cefoxitin (FOX), ceftriaxone (CRO), ceftazidime (CAZ), cefoperazone-sulbactam (CSL), cefepime (FEP), ertapenem (ETP), imipenem (IPM), tetracycline (TET), tigecycline (TGC), ciprofloxacin (CIP), chloramphenicol (CHL), gentamicin (GEN), tobramycin (TOB), amikacin (AMK), trimethoprim-sulfamethoxazole (SXT), and nitrofurantoin (NIT) were determined using a Vitek-2 instrument with VITEK-2 using AST *n*-101 and AST *n*-102 cards (BioMérieux, Paris, France). The results were interpreted according to the European Committee on Antimicrobial Susceptibility Testing (http://www.eucast.org/clinical_breakpoints, accessed on 1 November 2021). *E. coli* strains ATCC 25922 and ATCC 35218 were used for quality control. Strains non-susceptible to ≥1 agent in ≥3 antimicrobial categories were identified as multi-drug resistant (MDR), according to Magiorakos et al., 2012 [49].

### 4.4. Hypermucoviscousity Testing

The string test was used for the identification of hypermucoviscous *K. pneumoniae* strains growing on the plates with Luria-Bertani broth (Difco Laboratories, Detroit, MI, USA) overnight at 37 °C [23]. The positive test was assigned if a colony of *K. pneumoniae* formed viscous strings >5 mm length using a standard bacteriological loop.

### 4.5. K. pneumoniae Sequence Type and Capsular Type Identification

Sequence types (STs) of *K. pneumoniae* isolates were determined by the Multilocus Sequence Typing (MLST) scheme of Pasteur Institute (Paris, France) using the previously published primers [50,51]. The PCR capsular serotyping of the *K. pneumoniae* isolates was performed using specific primers for the *wzy* gene associated with K serotypes K1, K2, K20, and K57 [52] and by *wzi* gene sequencing for identification of capsular types K23, K27, K28, K31, K47, K60, K62, and K64 [53]. Bacterial thermolysates were used as DNA templates for amplification.

### 4.6. Detection of Antimicrobial Resistance and Virulence Genes

Beta-lactamase genes *bla*_SHV_, *bla*_CTX-M_, *bla*_TEM_, *bla*_OXA-48_, *bla*_KPC_, *bla*_VIM_, *bla*_IMP_, and *bla*_NDM_, class 1 and 2 integrons, and porin protein gene *ompK36*, as well as 8 genes associated with *K. pneumoniae* virulence, *rmpA* (hypermucoid phenotype regulator), *aer* (aerobactin), *kfu* (ferric absorption system), *uge* (uridine diphosphate-galacturonate-4-epimerase), *wabG* (glucosyltransferase), *fimH* (fimbria type I), *allS* and *allR* (allantoin regulon), were detected by PCR using specific primers as was described previously [10].

### 4.7. Real-Time PCR Detection of Antimicrobial Resistance and Virulence Genes

Oligonucleotides for RT-PCR (Table 2) were designed using Vector NTI Advance 11.0 (Invitrogen, Carlsbad, CA, USA) and purchased from Evrogen (Moscow, Russia). Secondary structures were controlled with Gene Runner 6.5.52 (http://www.generunner.net/, accessed on 1 November 2021) and in silico analysis by insilico.ehu.eus [54] was performed to check primer specificity. *K. pneumoniae* virulence genetic determinants (*rmpA*, *iroN*, *iroD*, *mrkD*, *uge*, *wabG*, *fimH*, *treC*, *celB*, *ureA*, and *allR*) and antibacterial resistance genes (*bla*_TEM_, *bla*_SHV_, *bla*_CTX-M_, *bla*_OXA-48_, *bla*_NDM_, *bla*_KPC_, *bla*_VIM_, *ompK36*) were detected by RT-PCR using qPCRmix-HS SYBR (Evrogen, Moscow, Russia) and the CFX96 Real-Time PCR system (Bio-Rad Laboratories, Hercules, CA, USA) with the following program: 95° for the 20s, 61° for 20s, 72° for 30 s, repeat 40 times. Bacterial thermolysates were used as DNA templates.

### 4.8. Whole-Genome Sequencing

WGS was carried out on the Illumina MiSeq platform using Nextera DNA Library Preparation Kit (Illumina, Carlsbad, CA, USA) and MiSeq Reagent Kits v3 (Illumina, Carlsbad, CA, USA). The obtained single reads were collected into contigs using the SPAdes 3.9.0 software (Petersburg State University, St-Petersburg, Russia). De novo assembled genomes were annotated in the GenBank database (https://github.com/ncbi/pgap, accessed on 1 November 2021). Antimicrobial resistance genes, STs, plasmids, and restriction-modification systems were identified using the web resource of the Center for Genomic Epidemiology (http://www.genomicepidemiology.org/, accessed on 1 November 2021). Virulence genes, capsular type, genes conferring resistance to heavy metals, and efflux pumps were identified by the Institut Pasteur, Paris, France, BIGS database web-resource of (https://bigsdb.pasteur.fr/, accessed on 1 November 2021).

### 4.9. K. pneumoniae Growing in Antimicrobial Conditions and RNA Isolation

Overnight bacterial culture was diluted 1/50 in Luria-Bertani broth (Difco Laboratories, Detroit, MI, USA) containing/not containing antimicrobials (100 mg/L AMP or 10 mg/L CRO) and incubated at 37 °C with agitation for 90 min. Each experiment was represented by three independent repeats. All following steps for RNA isolation were performed at 4 °C to limit RNase activity. Total RNA was isolated by phenol-chloroform extraction using kit RNA-extran (Sintol, Moscow, Russia), following manufacturer protocol. After isolation, RNA was treated with TURBO DNase (Invitrogen, Carlsbad, CA, USA) to remove traces of genomic DNA.

### 4.10. cDNA Synthesis and Quantitative Real-Time PCR

One µg of isolated total RNA was used for cDNA synthesis with RevertAid RT Reverse Transcription Kit (Thermo Fisher Scientific, Waltham, MA, USA). qPCR was performed using qPCRmix-HS SYBR (Evrogen, Moscow, Russia) and the CFX96 Real-Time PCR system (Bio-Rad Laboratories, Hercules, CA, USA) with the following program: 40 cycles of 20 s at 95 °C for denaturation, 20 s at 61 °C for annealing, 30 s at 72 °C for extension and SYBR Green detection. The melting curve analysis in the temperature range from 60 °C to 94 °C, with a fluorescence estimation step of 0.2 °C, was performed to confirm the specificity of the reaction. Relative quantification of the target gene expression was normalized with reference genes *proC*, *recA*, and *rpoD* expression. Three technical replicates per each of the three biological samples were used for statistical validity. The relative transcript levels of antimicrobial resistance and virulence genes were calculated using the 2^−ΔΔCt^ method [56]. A heat map of changes in gene expression levels relative to reference genes was designed using GraphPad Prism version 8.0.1 for Windows (GraphPad Software, La Jolla, CA, USA, www.graphpad.com accessed on 1 November 2021). Gene expression levels of each gene at present of AMP and CRO were compared to those in conditions without antimicrobials.

## Figures and Tables

**Table 1:** Characteristics of *K. pneumoniae* strains used for whole-genome sequence analysis.

Feature/strain	B185/19	B16K/19	B2523/18	B2580/14	B1261/19
SCPM-O	SCPM-O-B-9222	SCPM-O-B-9221	SCPM-O-B-9220	SCPM-O-B-7852	SCPM-O-B-9223
Isolation date	30-Jan-2019	09-Jan-2019	30-Aug-2018	21-Jul-2014	27-May-2019
Source	wound	urine	blood	urine	urine
GenBank	JAGVVR000000000.1	JAGVVS000000000.1	JAGRZF000000000.1	PUXF00000000.1	JAGVVQ000000000.1
Genome size, bp	5,812,311	5,532,115	5,430,135	5,773,846	5,570,452
Number of contigs	267	180	125	360	160
Genetic line	ST395^K47^	ST147^K64^	ST86^K2^	ST23^K1^	ST23^K57^
String-test	Negative	Negative	Positive	Positive	Positive
Antimicrobial resistance phenotype	AMP, SAM, CXM, FOX, CRO, CAZ, CSL, FEP, ETP, IPM, TET, CIP, CHL, GEN, TOB, AMK, SXT, NIT	AMP, SAM, CXM, FOX, CRO, CAZ, CSL, FEP, ETP, IPM, CIP, GEN, TOB, AMK, SXT, NIT	AMP	AMP, SAM, CXM, CRO, TET, CIP, CHL, TOB, SXT, NIT	AMP, SAM, CXM, FOX, CRO, CAZ, CSL, FEP, ETP, IPM, TET, TGC, CIP, CHL, GEN, TOB, SXT, NIT
Beta-lactamases	*bla*_SHV-12,_ *bla*_TEM-1B,_ *bla*_CTX-M-15,_ *bla*_OXA-1,_ *bla*_OXA-48,_ *bla*_NDM-1_	*bla*_SHV-67,_ *bla*_TEM-1B,_ *bla*_CTX-M-15,_ *bla*_OXA-1,_ *bla*_OXA-48,_ *bla*_NDM-1_	*bla*_SHV-28_	*bla*_SHV-190,_ *bla*_TEM-1B,_ *bla*_CTX-M-15,_ *bla*_OXA-1,_ *bla*_OXA-48_	*bla*_SHV-33,_ *bla*_CTX-M-55,_ *bla*_OXA-1,_ *bla*_OXA-48,_ *bla*_NDM-1_
AG-resistance	*aadA2, aph(3′)-Ia, armA*	*aph(3′)-VI, aadA1, aadA2, aadA5, armA*	*-*	*aph(6)-Id, aac(6′)-Ib-cr, aph(3″)-Ib*	*aph(6)-Id, aph(3′)-Ia, aph(3″)-Ib, aac(3)-IIa, aac(3)-IId, aadA16*
FOS-resistance	*fosA*	*fosA*	*fosA*	*fosA*	*fosA*
STR-resistance	*msr(E)*	*msr(E)*	*-*	*-*	
PHE-resistance	*catB3*	*catB3*	*-*	*catB3*	*catB3, floR*
QNL-resistance	*aac(6′)-Ib-cr, oqxA, oqxB*	*aac(6′)-Ib-cr, oqxA, oqxB*	*oqxA, oqxB*	*qnrB1, oqxA, oqxB*	*aac(6′)-Ib-cr, oqxA, oqxB*
SUL-resistance	*sul1*	*sul1*	*-*	*sul2*	*sul1, sul2*
TET-resistance	*tet(A)*	-	*-*	*tet(A)*	*tet(A)*
TRI-resistance	*dfrA1, dfrA12*	*dfrA12, dfrA17.*	*-*	*dfrA14*	*dfrA27*
MCR-resistance	*mph(A), mph(E)*	*mph(E)*	*-*	*-*	*mph(A)*
RIF-resistance	*-*	*-*	*-*	*-*	*ARR-3*
HM-resistance	*ars, pco, sil*	*sil*	*pbr, pco, sil, ter*	*pbr, pco, sil, ter*	*pbr, pco, sil, ter*
Efflux pums	*acr, oqx, kpn, kde, kex*	*acr, oqx, kpn, kde, kex*	*acr, oqx, kpn, kde, kex*	*acr, oqx, kpn, kde*	*acr, oqx, kpn, kde, kex*
Virulence genes	*uge, wabG, fimH, irp, iut, mrk, ybt, fyu,* *treC, cellB, ureA*	*uge, wabG, fimH, mrk,* *treC, cellB, ureA*	*peg-344, rmpA, iro, ent uge, wabG, fimH, irp, iuc, iut, mrk, ybt, fyu,* *treC, cellB, ureA*	*peg-344, rmpA, rmpA2, iro, ent, uge, wabG, kfu, fimH, allR, irp, iuc, iut, mrk, ybt, fyu,* *treC, cellB, ureA*	*peg-344, rmpA, ent, wabG, fimH, irp, iuc, iut, mrk, ybt, fyu,* *treC, cellB, ureA*
Plasmid	*ColRNAI, IncFIB(K), IncFII(K), IncR, IncX3*	*IncFIA(HI1), IncFIB(K), IncFIB, IncFII(K), IncHI1B, IncM2*	*IncHI1B, repB*	*IncFII(K), IncHI1B, IncL, repB*	*ColRNAI, IncC, IncFIA(HI1), IncFII, IncFII(K), IncL, repB*

Note: SCPM-O, State Collection of Pathogenic Microbes, AMP, ampicillin; SAM, ampicillin-sulbactam; CXM, cefuroxime; FOX, cefoxitin; CRO, ceftriaxone; CAZ, ceftazidime; CSL, cefoperazone-sulbactam; FEP, cefepime; ETP, ertapenem; IPM, imipenem; TET, tetracycline; TGC, tigecycline; CIP, ciprofloxacin; CHL, chloramphenicol; GEN, gentamicin; TOB, tobramycin; AMK, amikacin; SXT, trimethoprim-sulfamethoxazole; NIT, nitrofurantoin; AG, aminoglycosides; FOS, fosfomycin; STR, streptogramin; PHE, phenicol; QNL, quinolone; SUL, sulfonamide; TRI, trimethoprim; RIF, rifamycin; HM, heavy metal.

**Table 2:** Oligonucleotide primers for RT-PCR were used in this study for the detection of *K. pneumoniae* antimicrobial resistance and virulence genes.

Target Gene	Forward Primer (5′→3′)	Reverse Primer (5′→3′)	Size of PCR Product, bp
proC *	GATTGCCGATATCGTCTTCG	GAGACCACCAGCGACTCTTT	99
recA *	TTAAACAGGCCGAATTCCAG	CCGCTTTCTCAATCAGCTTC	99
rpoD *	TCCGGTGCATATGATTGAGA	ATACGCTCAGCCAGCTCTTC	105
*bla_TEM_*	GCCAACTTACTTCTGACAACGA	GATCAAGGCGAGTTACATG	88
*bla_SHV_*	GCCATTACCATGAGCGATAA	CAAAAAGGCAGTCAATCC	81
*bla_CTX-M_*	AAGCAGAGTGAAACGCAAAA	GGCAATCGGATTGTAGTTAA	84
*bla_OXA_-48*	GTGTTTTTGGTGGCATCGATTA	GGTTCGCCCGTTTAAGATTATT	164
*bla_NDM_*	ACTTTGGCCCGCTCAAGGTATT	TGATCAGGCAGCCACCAAAA	105
*ompK36*	CGGTTACGGCCAGTGGGAATA	GAGCCCGCGTCGCCAAATTT	111
*acrA*	ATGTGACGATAAACCGGCTC	CTGGCAGTTCGGTGGTTATT	101
*acrB*	TGGTGCTGCATGGCCAGGTT	CTTGTCCACCAGCCGCGAAA	149
*oqxA*	AGGCGCTTCCGGTGGAGATT	GCAAACAGCCCTGGCGTGAA	160
*oqxB*	CGCAACTACGCCACGCTGAA	GCCAGACGCGCATCGCATAT	103
*kpnE*	ATTGCTGAAATTACCGGCAC	AAATACCGATCCCTTCCCAC	172
*kpnF*	ATAACCCCCGCCCAGCCTTT	CCCTGTCGCAGGCGGTTAAA	139
*kexD*	TGAAGTTGCGCCACCCGGTA	TGTCGGTGCCGGGTTTGAAA	191
*kdeA*	CAGGCGCTGTTGTTCCCGTT	AAACATGCCGCCTGCCAGAT	162
*rmpA*	TGTACCTTTTGCAGCCAA	CTGCAATGCCTACTGAAAA	121
*iroN*	TTCCGGCTGGTTGGTGTATA	ATCGAAGTGATCCGTGGCC	132
*iroD*	ATAAGTTTGGCCGGCAGG	TTCGTGCCGAACAACCGC	183
*uge*	CCCGGATATGGCGCTGTTCA	CGCTTCATTTTGCCGTAGTT	84
*wabG*	AACGTATTCCCGGCTGCGATCT	CCGCGCAGGTGTGAGTCTTCAT	175
*fimH*	GCAGGATCAGCACCGCGATTAA	TCACGGACCGATAAACCCTGGC	106
*allR*	GAGCCTTCCGCGCAAAA	CCGCGCCTTCCAGCACTTTTA	163
*treC*	CCATTTCTCTCCGCCGGGATAA	ACCTGATGACCGTCGGCGAGAT	137
*celB*	GCGCGACGGCATCATTCT	GCGATGCCGAAGCTGGAAAT	192
*ureA*	GAGGGCGTCCCGGAAAT	TGTGAACGGTGACCAGCTT	79

Note: *, obtained from [55].

## Data Availability

The data presented in this study are openly available in the GenBank database at accession numbers [PUXF00000000.1, JAGRZF000000000.1, JAGVVS000000000.1, JAGVVR000000000.1, JAGVVQ000000000.1].

## Supplementary Materials

The following are available online at https://www.mdpi.com/article/10.3390/antibiotics11010007/s1, Table S1: Clinical characteristics, susceptibility to antibacterials, and antimicrobial resistance gene profiles of *K. pneumoniae* clinical isolates collected from 62 patients of Moscow Neuro-ICU, Table S2: *K. pneumoniae* virulence gene profiles of the clinical isolates collected from 62 patients of Moscow Neuro-ICU.

## References

[1] Bengoechea J.A., Sa Pessoa J. (2019). Klebsiella Pneumoniae Infection Biology: Living to Counteract Host Defences. FEMS Microbiol. Rev..

[2] Bialek-Davenet S., Criscuolo A., Ailloud F., Passet V., Jones L., Delannoy-Vieillard A.S., Garin B., le Hello S., Arlet G., Nicolas-Chanoine M.H. (2014). Genomic Definition of Hypervirulent and Multidrug-Resistant Klebsiella Pneumoniae Clonal Groups. Emerg. Infect. Dis..

[3] Baraniak A., Izdebski R., Fiett J., Sadowy E., Adler A., Kazma M., Salomon J., Lawrence C., Rossini A., Salvia A. (2013). Comparative Population Analysis of Klebsiella Pneumoniae Strains with Extended-Spectrum β-Lactamases Colonizing Patients in Rehabilitation Centers in Four Countries. Antimicrob. Agents Chemother..

[4] Struve C., Roe C.C., Stegger M., Stahlhut S.G., Hansen D.S., Engelthaler D.M., Andersen P.S., Driebe E.M., Keim P., Krogfelt K.A. (2015). Mapping the Evolution of Hypervirulent Klebsiella Pneumoniae. mBio.

[5] Choby J.E., Howard-Anderson J., Weiss D.S. (2020). Hypervirulent Klebsiella Pneumoniae—Clinical and Molecular Perspectives. J. Intern. Med..

[6] Xie M., Yang X., Xu Q., Ye L., Chen K., Zheng Z., Dong N., Sun Q., Shu L., Gu D. (2021). Clinical Evolution of ST11 Carbapenem Resistant and Hypervirulent Klebsiella Pneumoniae. Commun. Biol..

[7] Liu B.T., Su W.Q. (2019). Whole Genome Sequencing of NDM-1-Producing Serotype K1 St23 Hypervirulent Klebsiella Pneumoniae in China. J. Med. Microbiol..

[8] Turton J., Davies F., Turton J., Perry C., Payne Z., Pike R. (2019). Hybrid Resistance and Virulence Plasmids in “High-Risk” Clones of Klebsiella Pneumoniae, Including Those Carrying Blandm-5. Microorganisms.

[9] Ershova K., Savin I., Kurdyumova N., Wong D., Danilov G., Shifrin M., Alexandrova I., Sokolova E., Fursova N., Zelman V. (2018). Implementing an Infection Control and Prevention Program Decreases the Incidence of Healthcare-Associated Infections and Antibiotic Resistance in a Russian Neuro-ICU. Antimicrob. Resist. Infect. Control.

[10] Fursova N.K., Astashkin E.I., Ershova O.N., Aleksandrova I.A., Savin I.A., Novikova T.S., Fedyukina G.N., Kislichkina A.A., Fursov M.V., Kuzina E.S. (2021). Multidrug-Resistant Klebsiella Pneumoniae Causing Severe Infections in the Neuro-Icu. Antibiotics.

[11] Jain N., Jansone I., Obidenova T., Sīmanis R., Meisters J., Straupmane D., Reinis A. (2021). Epidemiological Characterization of Clinical Fungal Isolates from Pauls Stradinš Clinical University Hospital, Latvia: A 4-Year Surveillance Report. Life.

[12] Wang L., Zhou K.H., Chen W., Yu Y., Feng S.F. (2019). Epidemiology and Risk Factors for Nosocomial Infection in the Respiratory Intensive Care Unit of a Teaching Hospital in China: A Prospective Surveillance during 2013 and 2015. BMC Infect. Dis..

[13] Fursova N.K., Astashkin E.I., Knyazeva A.I., Kartsev N.N., Leonova E.S., Ershova O.N., Alexandrova I.A., Kurdyumova N.V., Sazikina S.Y., Volozhantsev N.V. (2015). The spread of bla OXA-48 and bla OXA-244 carbapenemase genes among Klebsiella pneumoniae, Proteus mirabilis and Enterobacter spp. isolated in Moscow, Russia. Ann. Clin. Microbiol. Antimicrob..

[14] Izdebski R., Baraniak A., Zabicka D., Machulska M., Urbanowicz P., Fiett J., Literacka E., Bojarska K., Kozińska A., Zieniuk B. (2018). Enterobacteriaceae Producing OXA-48-like Carbapenemases in Poland, 2013-January 2017. J. Antimicrob. Chemother..

[15] Muggeo A., Guillard T., Klein F., Reffuveille F., François C., Babosan A., Bajolet O., Bertrand X., de Champs C. (2018). Spread of Klebsiella Pneumoniae ST395 Non-Susceptible to Carbapenems and Resistant to Fluoroquinolones in North-Eastern France. J. Glob. Antimicrob. Resist..

[16] Maida C.M., Bonura C., Geraci D.M., Graziano G., Carattoli A., Rizzo A., Torregrossa M.V., Vecchio D., Giuffrè M. (2018). Outbreak of ST395 KPC-Producing Klebsiella Pneumoniae in a Neonatal Intensive Care Unit in Palermo, Italy. Infect. Control Hosp. Epidemiol..

[17] Fursova N.K., Astashkin E.I., Gabrielyan N.I., Novikova T.S., Fedyukina G.N., Kubanova M.K., Esenova N.M., Sharapchenko S.O., Volozhantsev N.V. (2020). Emergence of Five Genetic Lines ST395NDM-1, ST13OXA-48, ST3346OXA-48, ST39CTX-M-14, and Novel ST3551OXA-48of Multidrug-Resistant Clinical Klebsiella Pneumoniae in Russia. Microb. Drug Resist..

[18] Peirano G., Chen L., Kreiswirth B.N., Pitout J.D.D. (2020). Emerging Antimicrobial-Resistant High-Risk Klebsiella Pneumoniae Clones ST307 and ST147. Antimicrob. Agents Chemother..

[19] Shankar C., Jacob J.J., Vasudevan K., Biswas R., Manesh A., Sethuvel D.P.M., Varughese S., Biswas I., Veeraraghavan B. (2020). Emergence of Multidrug Resistant Hypervirulent ST23 Klebsiella Pneumoniae: Multidrug Resistant Plasmid Acquisition Drives Evolution. Front. Cell. Infect. Microbiol..

[20] Baron S.A., Pascale L.M., Million M., Briantais A., Durand J.M., Hadjadj L., Rolain J.M. (2021). Whole Genome Sequencing to Decipher the Virulence Phenotype of Hypervirulent Klebsiella Pneumoniae Responsible for Liver Abscess, Marseille, France. Eur. J. Clin. Microbiol. Infect. Dis..

[21] Liao C.H., Huang Y.T., Chang C.Y., Hsu H.S., Hsueh P.R. (2014). Capsular Serotypes and Multilocus Sequence Types of Bacteremic Klebsiella Pneumoniae Isolates Associated with Different Types of Infections. Eur. J. Clin. Microbiol. Infect. Dis..

[22] Lev A.I., Astashkin E.I., Kislichkina A.A., Solovieva E.V., Kombarova T.I., Korobova O.V., Ershova O.N., Alexandrova I.A., Malikov V.E., Bogun A.G. (2018). Comparative Analysis of Klebsiella Pneumoniae Strains Isolated in 2012–2016 That Differ by Antibiotic Resistance Genes and Virulence Genes Profiles. Pathog. Glob. Health.

[23] Shon A.S., Bajwa R.P.S., Russo T.A. (2013). Hypervirulent (Hypermucoviscous) Klebsiella Pneumoniae: A New and Dangerous Breed. Virulence.

[24] Zhang Y., Jin L., Ouyang P., Wang Q., Wang R., Wang J., Gao H., Wang X., Wang H. (2020). Evolution of Hypervirulence in Carbapenem-Resistant Klebsiella Pneumoniae in China: A Multicentre, Molecular Epidemiological Analysis. J. Antimicrob. Chemother..

[25] Wyres K.L., Lam M.M.C., Holt K.E. (2020). Population Genomics of Klebsiella Pneumoniae. Nat. Rev. Microbiol..

[26] Xie M., Dong N., Chen K., Yang X., Ye L., Chan E.W.C., Zhang R., Chen S. (2020). A Hybrid Plasmid Formed by Recombination of a Virulence Plasmid and a Resistance Plasmid in Klebsiella Pneumoniae. J. Glob. Antimicrob. Resist..

[27] Yuan Y., Li Y., Wang G., Li C., Chang Y.F., Chen W., Nian S., Mao Y., Zhang J., Zhong F. (2019). Bla NDM-5 Carried by a Hypervirulent Klebsiella Pneumoniae with Sequence Type 29. Antimicrob. Resist. Infect. Control.

[28] Mohammad Ali Tabrizi A., Badmasti F., Shahcheraghi F., Azizi O. (2018). Outbreak of Hypervirulent Klebsiella Pneumoniae Harbouring BlaVIM-2 among Mechanically-Ventilated Drug-Poisoning Patients with High Mortality Rate in Iran. J. Glob. Antimicrob. Resist..

[29] Lazareva I., Ageevets V., Sopova J., Lebedeva M., Starkova P., Likholetova D., Lebedeva M., Gostev V., Moiseenko V., Egorenkov V. (2020). The Emergence of Hypervirulent BlaNDM-1-Positive Klebsiella Pneumoniae Sequence Type 395 in an Oncology Hospital. Infect. Genet. Evol..

[30] Hou X.H., Song X.Y., Ma X.B., Zhang S.Y., Zhang J.Q. (2015). Molecular Characterization of Multidrug-Resistant Klebsiella Pneumoniae Isolates. Braz. J. Microbiol..

[31] Farhadi M., Ahanjan M., Goli H.R., Haghshenas M.R., Gholami M. (2021). High Frequency of Multidrug-Resistant (MDR) Klebsiella Pneumoniae Harboring Several β-Lactamase and Integron Genes Collected from Several Hospitals in the North of Iran. Ann. Clin. Microbiol. Antimicrob..

[32] Lin Z.W., Zheng J.X., Bai B., Xu G.J., Lin F.J., Chen Z., Sun X., Qu D., Yu Z.J., Deng Q.W. (2020). Characteristics of Hypervirulent Klebsiella Pneumoniae: Does Low Expression of RmpA Contribute to the Absence of Hypervirulence?. Front. Microbiol..

[33] Protonotariou E., Meletis G., Chatzopoulou F., Malousi A., Chatzidimitriou D., Skoura L. (2019). Emergence of Klebsiella Pneumoniae ST11 Co-Producing NDM-1 and OXA-48 Carbapenemases in Greece. J. Glob. Antimicrob. Resist..

[34] Hernández M., López-Urrutia L., Abad D., Serna M.D.F., Ocampo-Sosa A.A., Eiros J.M. (2021). First Report of an Extensively Drug-Resistant ST23 Klebsiella Pneumoniae of Capsular Serotype K1 Co-Producing CTX-M-15, OXA-48 and ArmA in Spain. Antibiotics.

[35] Carvalho I., Carvalho J.A., Martínez-álvarez S., Sadi M., Capita R., Alonso-Calleja C., Rabbi F., de Lurdes Nunes Enes Dapkevicius M., Igrejas G., Torres C. (2021). Characterization of Esbl-Producing Escherichia Coli and Klebsiella Pneumoniae Isolated from Clinical Samples in a Northern Portuguese Hospital: Predominance of Ctx-m-15 and High Genetic Diversity. Microorganisms.

[36] Chaves J., Ladona M.G., Segura C., Coira A., Reig R., Ampurdanés C. (2001). SHV-1 β-Lactamase Is Mainly a Chromosomally Encoded Species-Specific Enzyme in Klebsiella Pneumoniae. Antimicrob. Agents Chemother..

[37] Awosile B.B., Agbaje M. (2021). Genetic Environments of Plasmid-Mediated BlaCTXM-15 Beta-Lactamase Gene in Enterobacteriaceae from Africa. Microbiol. Res..

[38] Barguigua A., el Otmani F., Talmi M., Reguig A., Jamali L., Zerouali K., Timinouni M. (2013). Prevalence and Genotypic Analysis of Plasmid-Mediated β-Lactamases among Urinary Klebsiella Pneumoniae Isolates in Moroccan Community. J. Antibiot..

[39] Shi W., Qin J., Mi Z. (2008). A Klebsiella pneumoniaesputum culture isolate from China carrying blaOXA-1, blaCTX-M-55 and aac(69)-Ib-cr. J. Med. Microbiol..

[40] Sherchan J.B., Tada T., Shrestha S., Uchida H., Hishinuma T., Morioka S., Shahi R.K., Bhandari S., Twi R.T., Kirikae T. (2020). Emergence of Clinical Isolates of Highly Carbapenem-Resistant Klebsiella Pneumoniae Co-Harboring BlaNDM-5 and BlaOXA-181 or -232 in Nepal. Int. J. Infect. Dis..

[41] Maurya N., Jangra M., Tambat R., Nandanwar H. (2019). Alliance of Efflux Pumps with β-Lactamases in Multidrug-Resistant Klebsiella Pneumoniae Isolates. Microb. Drug Resist..

[42] Mirzaie A., Ranjbar R. (2021). Antibiotic Resistance, Virulence-Associated Genes Analysis and Molecular Typing of Klebsiella Pneumoniae Strains Recovered from Clinical Samples. AMB Express.

[43] Russo T.A., Olson R., Fang C.T., Stoesser N., Miller M., MacDonald U., Hutson A., Barker J.H., la Hoz R.M., Johnson J.R. (2018). Identification of Biomarkers for Differentiation of Hypervirulent Klebsiella Pneumoniae from Classical K. Pneumoniae. J. Clin. Microbiol..

[44] Russo T.A., MacDonald U., Hassan S., Camanzo E., LeBreton F., Corey B., McGann P. (2021). An Assessment of Siderophore Production, Mucoviscosity, and Mouse Infection Models for Defining the Virulence Spectrum of Hypervirulent Klebsiella Pneumoniae. mSphere.

[45] Shaidullina E., Shelenkov A., Yanushevich Y., Mikhaylova Y., Shagin D., Alexandrova I., Ershova O., Akimkin V., Kozlov R., Edelstein M. (2020). Antimicrobial Resistance and Genomic Characterization of OXA-48-and CTX-M-15-Co-Producing Hypervirulent Klebsiella Pneumoniae St23 Recovered from Nosocomial Outbreak. Antibiotics.

[46] Abdullah F., Qarasnji B.K., Abdullah Tawgozy F.H., Amin B.K. (2016). Molecular Study of SHV-11 and SHV-12 Genes among Klebsiella Pneumoniae Isolated from UTI Patients in Erbil City. Zanco J. Pure Appl. Sci..

[47] Pal A., Dhara L., Tripathi A. (2019). Contribution of AcrB Upregulation & OmpC/Ompk36 Loss over the Presence of BlaNDM towards Carbapenem Resistance Development among Pathogenic Escherichia Coli & Klebsiella Spp.. Indian J. Med. Res..

[48] Hennequin C., Robin F. (2016). Correlation between Antimicrobial Resistance and Virulence in Klebsiella Pneumoniae. Eur. J. Clin. Microbiol. Infect. Dis..

[49] Magiorakos A.P., Srinivasan A., Carey R.B., Carmeli Y., Falagas M.E., Giske C.G., Harbarth S., Hindler J.F., Kahlmeter G., Olsson-Liljequist B. (2012). Multidrug-Resistant, Extensively Drug-Resistant and Pandrug-Resistant Bacteria: An International Expert Proposal for Interim Standard Definitions for Acquired Resistance. Clin. Microbiol. Infect..

[50] Diancourt L., Passet V., Verhoef J., Grimont P.A.D., Brisse S. (2005). Multilocus Sequence Typing of Klebsiella Pneumoniae Nosocomial Isolates. J. Clin. Microbiol..

[51] Brisse S., Fevre C., Passet V., Issenhuth-Jeanjean S., Tournebize R., Diancourt L., Grimont P. (2009). Virulent Clones of Klebsiella Pneumoniae: Identification and Evolutionary Scenario Based on Genomic and Phenotypic Characterization. PLoS ONE.

[52] Fang C.T., Lai S.Y., Yi W.C., Hsueh P.R., Liu K.L., Chang S.C. (2007). Klebsiella Pneumoniae Genotype K1: An Emerging Pathogen That Causes Septic Ocular or Central Nervous System Complications from Pyogenic Liver Abscess. Clin. Infect. Dis..

[53] Brisse S., Passet V., Haugaard A.B., Babosan A., Kassis-Chikhani N., Struve C., Decre D. (2013). Wzi Gene Sequencing, a Rapid Method for Determination of Capsulartype for Klebsiella Strains. J. Clin. Microbiol..

[54] Bikandi J., Millán R.S., Rementeria A., Garaizar J. (2004). In Silico Analysis of Complete Bacterial Genomes: PCR, AFLP-PCR and Endonuclease Restriction. Bioinformatics.

[55] Gomes A.É.I., Stuchi L.P., Siqueira N.M.G., Henrique J.B., Vicentini R., Ribeiro M.L., Darrieux M., Ferraz L.F.C. (2018). Selection and Validation of Reference Genes for Gene Expression Studies in Klebsiella Pneumoniae Using Reverse Transcription Quantitative Real-Time PCR. Sci. Rep..

[56] Livak K.J., Schmittgen T.D. (2001). Analysis of Relative Gene Expression Data Using Real-Time Quantitative PCR and the 2-ΔΔCT Method. Methods.

